# Hepatitis E virus infection and renal injury in non-immunocompromised host: clinical investigation and rabbit model study

**DOI:** 10.3389/fcimb.2025.1583006

**Published:** 2025-05-06

**Authors:** Weigang Zhang, Qiyu He, Hao Wang, Wanyun Gong, Lei Qin, Yunjie Lu, Yuting Wang, Wei Chen, Ling Wang, Wei He, Lin Wang, Yu Wang

**Affiliations:** ^1^ Department of General Surgery, First Affiliated Hospital of Soochow University, Suzhou, Jiangsu, China; ^2^ Department of Microbiology and Infectious Disease Center, School of Basic Medical Sciences, Peking University Health Science Center, Beijing, China; ^3^ Department of General Surgery, Children’s Hospital of Nanjing Medical University, Nanjing, China; ^4^ Department of Infectious Diseases, Kunshan First People’s Hospital Affiliated to Jiangsu University, Kunshan, Jiangsu, China; ^5^ Department of General Surgery, Wujin Affiliated Hospital of Jiangsu University and Wujin Clinical College of Xuzhou Medical University, Changzhou, Jiangsu, China; ^6^ Department of Hepatobiliary Surgery, Jintan Affiliated Hospital of Jiangsu University, Changzhou, Jiangsu, China

**Keywords:** hepatitis E, kidney, clinical investigation, rabbit model, HEV (hepatitis E virus)

## Abstract

**Background:**

Hepatitis E virus (HEV)-associated renal injury is mainly reported in immunocompromised patients. Here we investigated HEV-associated renal injury in non-immunocompromised acute hepatitis E (AHE) patients and rabbits.

**Methods:**

A total of 35 non-immunocompromised AHE patients were tested for kidney function parameters and HEV markers. HEV3- and HEV4-infected rabbits were tested for alanine aminotransferase, creatinine (Cr), and HEV markers. HEV-associated renal injury and renal HEV replication were analyzed by histopathology and RT-qPCR.

**Results:**

The non-immunocompromised AHE patients all showed normal serum Cr, blood urea nitrogen (BUN), and urine acid (UA). However, 25% of non-immunocompromised AHE patients showed proteinuria. In the rabbit model, HEV replication was observed in kidney tissues. The HEV-infected rabbits showed a transient elevated Cr level. Renal injury, including focal lymphocytic infiltration and tubular protein casts, was observed in rabbits across acute, recovery, and chronic phases of HEV infection.

**Conclusions:**

Proteinuria is not uncommon in non-immunocompromised AHE patients, indicating that HEV infection affects the kidney. We further proved that HEV can cause renal injury in a rabbit model.

## Introduction

Hepatitis E virus (HEV) is the causative agent of acute viral hepatitis worldwide (Kamar et al., 2017; Nimgaonkar et al., 2018). There are four major human-pathogenic HEV genotypes (HEV1–4), which show different geographical distribution and transmission patterns. HEV1 and HEV2 are waterborne pathogens mainly endemic to tropical regions with inadequate sanitation, whereas HEV3 and HEV4 exhibit zoonotic transmission through the consumption of contaminated meat, accounting for sporadic cases in developed countries and China (Kamar et al., 2017; Nimgaonkar et al., 2018). HEV infection is generally self-limiting, but chronic hepatitis E has been reported in immunocompromised individuals, such as solid organ transplant recipients (Kamar et al., 2008a), HIV/AIDS individuals (Dalton et al., 2009), and cancer patients (von Felden et al., 2019).

While HEV primarily replicates in the liver and induces acute hepatitis E (AHE), recent studies demonstrate the presence of HEV RNA and/or antigens in extra-hepatic tissues, such as the gut, cerebrospinal fluid, bone marrow, and kidneys across human or animal models (Williams et al., 2001; Geng et al., 2016; Wang et al., 2017; Fritz et al., 2018; Wang et al., 2020). HEV-associated extra-hepatic manifestations have been frequently reported, especially renal injury (Kamar et al., 2012; Pischke et al., 2017). Clinical studies have documented significant reductions in estimated glomerular filtration rate (eGFR) in HEV-infected kidney and liver transplant patients, with multiple case reports establishing a strong association between HEV and glomerular diseases (Ali et al., 2001; Kamar et al., 2012; Taton et al., 2013; Del et al., 2015; Guinault et al., 2016). In these cases, glomerulonephritis is commonly reported, with membranoproliferative glomerulonephritis, IgA nephropathy, and membranous glomerulonephritis being the most frequent subtypes identified through kidney biopsy (Gerolami et al., 2008; Kamar et al., 2008a; Kamar et al., 2008b; Taton et al., 2013; Del et al., 2015; Guinault et al., 2016; Leblond et al., 2024). Most of the HEV-associated renal manifestations occurred and were reported in immunocompromised individuals (Pischke et al., 2017), while the kidney function parameters may be frequently overlooked during the clinical management of non-immunocompromised AHE patients. Moreover, the dynamics of kidney function parameters and HEV replication and the detailed renal pathology progression during HEV infection remain to be fully elucidated in an *in vivo* model.

To date, our understanding of HEV-associated renal injury and the different prognosis between the liver and the kidney following HEV infection remains limited. This study aims to investigate HEV-associated renal injury from both aspects of clinical and animal study.

## Methods

### Patient cohorts and sample collection

AHE patients were retrospectively enrolled from Kunshan First People’s Hospital affiliated to Jiangsu University and Jintan Affiliated Hospital of Jiangsu University, Jiangsu Province, China, from January 2022 to May 2024. Their liver and kidney function parameters were analyzed. AHE was diagnosed by clear symptoms of hepatitis and elevation of liver enzyme levels on admission. HEV infection was diagnosed with anti-HEV IgM and/or a spontaneous rise of anti-HEV IgG (EASL Clinical Practice Guidelines on hepatitis E virus infection, 2018). All of the recruited patients were also tested for hepatitis A, B, and C virus, respectively, Epstein–Barr virus, and cytomegalovirus infection. The collected samples from AHE patients on admission were immediately sent to our laboratory where they were processed as previously described (Wang et al., 2016). All of the patients gave informed consent and permission for clinical records and testing of clinical samples. The Ethics Committee of Jintan Affiliated Hospital of Jiangsu University approved the study (No. 2023007).

### Animals

The animal experiments were approved by the Committee of Laboratory Animal Welfare and Ethics, Peking University Health Science Center. A total of 24 3-month-old New Zealand white rabbits, weighing 2.5–3 kg, were randomly selected from Beijing Jinmuyang Laboratory Animal Breeding Co., Ltd. Serum and fecal samples were collected weekly. All of the samples were found negative for anti-HEV antibodies by an enzyme-linked immunosorbent assay (ELISA) and HEV RNA by a previously described reverse transcription-nested polymerase chain reaction (RT-nPCR) and quantitative reverse transcription PCR (RT-qPCR) (Li et al., 2019).

### Virus inoculum

The rabbit HEV3 (HEV-3ra) strain CHN-BJ-R14 (GenBank number: JX109834) used in this study was recovered from the fecal samples of a farmed rabbit in Beijing. The swine HEV4 strain CHN-SD-SW2 (GenBank number: KP284140) was first recovered from pigs (Xia et al., 2015) and adapted in laboratory rabbits (Zhang et al., 2015). The fecal samples were diluted in phosphate-buffered saline to make a 10% (wt./vol) suspension and centrifuged at 4,000 rpm at 4°C for 30 min. The clarified suspension was filtered through 0.45- and 0.22-μm filters and titrated subsequently. The titer of the resulting positive inoculum was about 3 × 10^6^ copies/mL. The negative inoculum was prepared from the fecal samples of a non-HEV-infected rabbit.

### Experimental design of the animal study

All of the rabbits were randomly divided into three groups, designated as group A (*n* = 10), group B (*n* = 11), and group C (*n* = 3). The rabbits in group A and group B were inoculated intravenously with 1 mL of HEV-3ra-positive or HEV4-positive inoculum, respectively, while the rabbits in group C were inoculated intravenously with 1 mL of negative inoculum as a negative control. Three rabbits each in group A and B were euthanized for necropsy at 1 week after Cr was elevated during the acute phase of HEV infection. Three other rabbits each in groups A and B were euthanized for necropsy at the recovery phase of HEV infection, which is defined as the first week when fecal virus shedding stopped. The remaining rabbits were monitored until the end of the infection or the study, of which one rabbit in group A developed chronic HEV-3ra infection and was euthanized for necropsy at 32 wpi at the chronic phase of HEV infection. The end of the infection was defined as the time when fecal virus shedding was undetectable for 3 consecutive weeks. The study ended at 32 wpi when the rabbit with chronic HEV-3ra infection was euthanized.

### Sample collection and processing in the animal study

Serum and fecal samples were collected weekly and stored at -80°C. The serum samples were tested for ALT and Cr through standard methods. The fecal samples were processed as previously described and tested for HEV RNA (Li et al., 2019). After euthanizing the rabbits, partial kidney and liver specimens were taken and stored at -80°C for HEV RNA detection, and the rest was fixed in neutral 4% paraformaldehyde for 3 days for histopathological analysis. Furthermore, 100 mg of the tissue was homogenized in 1 ml of TRIzol reagent (Invitrogen, Burlington, ON, Canada) and clarified by centrifugation at 12,000 rpm at 4°C for 15 min. The supernatant was harvested and stored at -80°C for detection of HEV RNA and viral load. To prevent cross-contamination during necropsy, disposable sterile materials and new sterile scalpel blades were used independently for each sample.

### Determination of ALT and Cr concentrations in the animal study

The serum samples of the rabbits were tested for serum ALT and Cr concentrations using standard methods on Hitachi Automatic Clinical Analyzer 7180. To determine the normal ranges of the abovementioned parameters, serum samples collected from 10 healthy rabbits were analyzed. The rabbits were considered to have liver injury or kidney function impairment when the serum ALT or Cr concentration exceeded the pre-challenge level by more than twofold.

### Detection of HEV RNA

Total RNA was extracted from 100 μL of fecal suspensions or 500 μL supernatants of tissue homogenates using TRIzol reagent (Invitrogen, Burlington, ON, Canada) following a standard instruction. HEV viral load was determined using a commercial one-step real-time quantitative PCR assay (GoTaq^®^ Probe 1-step RT-qPCR System Kit, Promega, WI, USA) with the forward primer (JVHEVF, 5′-GGTGGTTTCTGGGGTGAC-3′), the reverse primer (JVHEVR, 5′- AGGGGTTGGTTGGATGAA-3′), and a hybridization probe (JVHEVP, 5′FAM- TGATTCTCAGCCCTTCGC-3′BHQ1) as previously described (Li et al., 2019).

### Histopathology

The tissue and organ samples were fixed in 10% neutral buffered formalin, embedded in paraffin, and cut into 5-μM serial sections. The slides were stained with hematoxylin and eosin (HE). The samples were photographed and analyzed under a microscope (Olympus CX31, Japan) equipped with a digital camera with the assistance of Motic Images Plus 2.0 software.

### Statistical analysis

Statistical analysis was performed with IBM SPSS Statistics 26.0 software (SPSS Inc., Chicago, IL, USA). Quantitative variables were compared by either one-way ANOVA test and followed by LSD test or Student’s *t*-test. The proportions in different sub-groups were compared by chi-square test or Fisher’s exact test. *P <*0.05 was considered to indicate statistical significance.

## Results

### Laboratory examinations of non-immunocompromised patients with AHE

The enrolled AHE patients included 19 male and 14 female individuals, respectively. The patients’ age varied from 38 to 82 years old (56.8 ± 13.5). They neither receive any immunosuppressants and immunoregulators nor had a disease that could affect their immune system (such as HIV infection or tumor). They are diagnosed with HEV infection with anti-HEV IgM and/or a spontaneous rise of anti-HEV IgG (**Table 1**). The results of the liver function test and the kidney function test for these non-immunocompromised patients are listed in **Table 1**. The serum ALT, AST, and Tbil levels in the AHE cohorts were 369.0 ± 862.9, 331.0 ± 908.2, and 44.7 ± 37.3, respectively. The Cr, blood urea nitrogen (BUN), and urine acid (UA) levels in the AHE cohorts were 62.1 ± 15.7, 4.6 ± 1.2, and 296.8 ± 81.1, respectively. Except for one patient who showed a slightly higher UA level (UA: 478 μmol/L), all of the AHE patients showed normal serum Cr, BUN, and UA levels on admission. A total of 28 patients were tested for proteinuria. Surprisingly, as high as 25% (7/28) of AHE patients showed proteinuria.

**Table 1:** Laboratory examinations of non-immunocompromised patients with acute hepatitis E.

Patients	Sex	Age	Liver function				Kidney function				HEV marker	
			ALT (9–50 U/L)	AST (15–40 U/L)		Tbil (≤26.0 μmol/L)	Cr (57–97 μmol/L)	BUN (3.1–8.0 mmol/L)	UA (208–428 μmol/L)	Proteinuria	IgM	IgG
1	F	37	550	171		115	47	3.9	211	–	+	NA
2	F	59	86	26		14.3	41	4.2	349	(+)	+	NA
3	M	59	50	17		18.3	93	6.2	410	–	57.598	7.907
4	M	47	349	39		15.1	85	4.9	393	–	8.822	4.04
5	M	43	38	19		23.5	109	6.3	478	(++)	10.168	10.329
6	M	50	105	30		29.5	51	3.5	302	–	2.609	1.968
7	F	51	19	14		35	27	5	280	(+)	50.333	35.525
8	F	50	23	27		25.6	52	3.8	406	–	2.609	1.968
9	F	68	98	132		144	54	6.7	343	–	1.122	6.858
10	M	41	43	24		21.3	73	3.8	307	–	7.157	9.935
11	M	38	46	18		10.9	64	3.9	346	NA	1.152	0.492
12	M	52	42	19		17.8	60	4.1	289	–	1.853	6.67
13	M	47	127	67		85.9	78	3.1	285	–	50.582	13.26
14	F	67	17	24		23	57	4.3	201	–	16.294	26.968
15	M	51	31	25		68.3	57	6.3	195	–	61.305	19.8
16	F	43	57	28		28.2	49	3.2	403	NA	+	NA
17	M	73	43	45		44.2	63	3.5	268	–	31.181	16.409
18	M	59	67	20		22.3	64	4.5	329	–	9.806	15.66
19	F	53	92	25		19.4	49	8.3	168	–	31.372	11.301
20	F	82	67	78		50.4	72	7.1	261	(+-)	12.468	20.093
21	M	70	56	26		13.1	81	3.7	319	–	1.867	7.057
22	F	38	78	29		18.7	52	4.3	273	(+)	34.894	0.155
23	M	58	984	1116		67	61	2.9	139	–	23.606	18.051
24	M	68	48	63		68.8	63	4.1	272	–	3.472	31.818
25	F	57	43	16		10.1	42	4.7	170	(+)	7.748	3.029
26	F	61	26	52		8.4	55	3.4	197	NA	2.632	9.349
27	F	61	94	49		25.7	53	5.4	276	(+-)	26.895	NA
28	M	40	67	59		47.7	64	3.4	413	–	23.041	0.449
29	M	76	74	59		61.2	60	4.3	247	–	51.331	18.825
30	M	40	4291	4329		114.2	61	5	420	NA	+	NA
31	M	82	2547	3269		163.2	73	4.74	257.3	NA	+	NA
32	M	77	1812	770		87.1	71	4.63	297	NA	+	NA
33	F	77	515	667		111.5	79	6.59	328.5	NA	+	NA
											

### Dynamics of liver and kidney function in the HEV-infected rabbit model

In order to assess the dynamic change of HEV-associated renal injury, we established HEV3-infected (group A) and HEV4-infected (group B) rabbit models. Group C was set up as a negative control. The kidney function indicator serum Cr concentrations were monitored weekly. The rabbits in the three groups were euthanized for necropsy at the acute phase (4 or 5 wpi) and at the recovery phase (when fecal HEV RNA was negative). One rabbit in group A developed chronic HEV infection and was monitored until 32 wpi.

**Figure 1:**
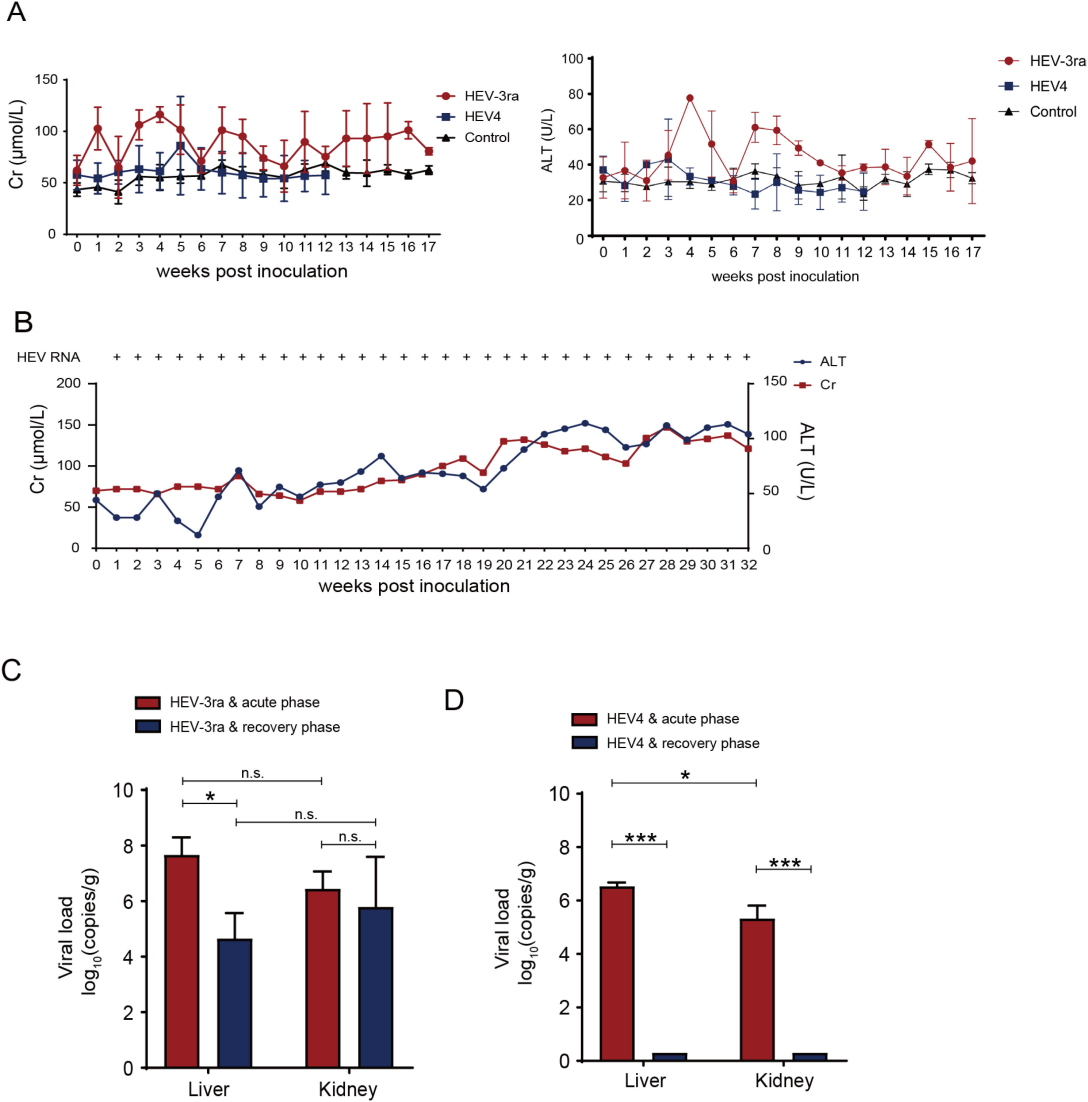
Dynamics of liver and kidney function in the HEV-infected rabbit model. **(A)** Serum creatinine (Cr) and alanine aminotransferase (ATL) of rabbits with virus spontaneous clearance in group A and B inoculated with rabbit HEV3 (HEV-3ra) and HEV4, respectively. **(B)** Serum Cr and ATL concentrations and detection of HEV RNA of the rabbit which had chronic HEV-3ra infection for over 6 months and was euthanized at 32 weeks post-infection in group **(A, C)** Viral loads in liver and kidney tissues of rabbits at the acute and recovery phases of HEV-3ra. **(D)** Viral loads in the liver and kidney tissues of rabbits at the acute and recovery phases of HEV4 infection. Data are means ± SD. *P < 0.05; ***P < 0.001; n.s., not significant.

The levels of ALT and Cr had a transient increase and reached the peak at 4 wpi in rabbits with acute infection in group A. In group B rabbits, no obvious increase of ALT level was observed, while the Cr level had a slight increase at 5 wpi (**Figure 1A**). In the rabbit with chronic HEV infection, the ALT and Cr concentrations peaked at 24 and 28 wpi, respectively, both exceeding more than twofold above their baseline values (**Figure 1B**). The Cr level of almost all infected rabbits changed synchronously with the ALT level and returned to the baseline level when the infection was cleared. The rabbits in both groups A and B were seroconverted for anti-HEV antibodies. The rabbits in control group C had normal ALT and Cr levels and remained negative for HEV RNA and anti-HEV antibodies throughout this investigation.

### HEV replication in the kidney of experimentally infected rabbits

Viral RNA loads were detected in both liver and kidney tissues at the acute phase of HEV-3ra and HEV4 infection, respectively (**Figures 1C,D**). In group A, the viral RNA loads of liver tissues were significantly decreased (*P* = 0.010) at the recovery phase compared with that at the acute phase. However, no significant decrease of viral RNA loads was observed in the kidney at the recovery phase compared with that at the acute phase (**Figure 1C**). In group B, viral RNA loads were significantly higher in the liver than in the kidney at the acute phase, and both tissues were detected to be negative for HEV RNA at the recovery phase (**Figure 1D**). The liver and kidney tissues of the rabbits in control group C were detected to be negative for HEV RNA.

### Histopathology analysis of kidney tissue in experimentally infected rabbits

**Figure 2:**
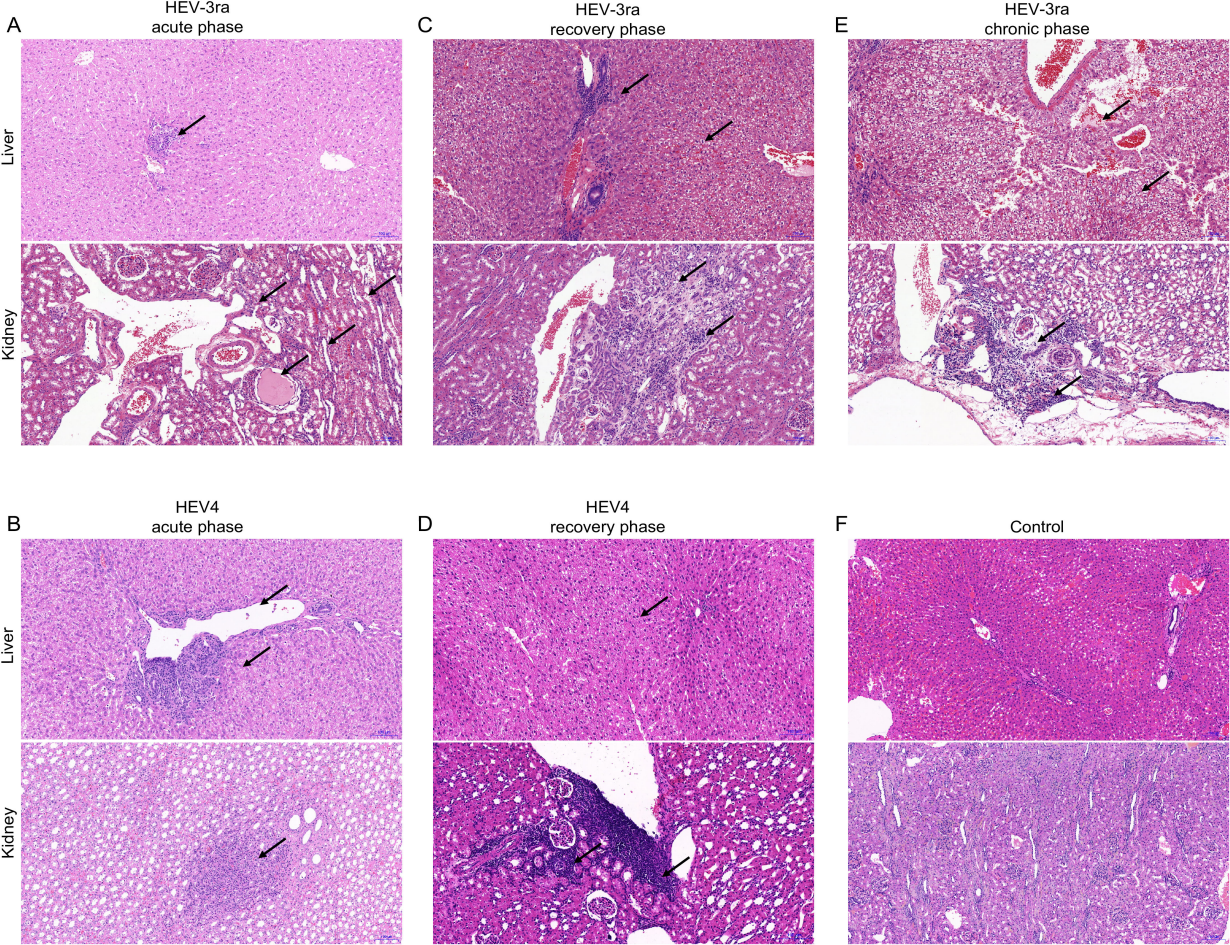
Histopathology of kidney and liver in rabbits with acute and chronic hepatitis E virus (HEV) infection. HE staining of liver and kidney sections from rabbits at the acute and recovery phases of rabbit HEV3 (HEV-3ra) or HEV4 infection, with chronic HEV-3ra infection, and in the control group. The arrows indicate representative pathological injury. **(A, B)** The rabbits at the acute phase of HEV-3ra or HEV4 infection showed hepatocyte degeneration and necrosis, collagen fiber hyperplasia, and fibrosis in the liver section. Focal lymphocytic infiltration and protein casts in the renal tubular lumen were seen in the kidney section. **(C, D)** The rabbits at the recovery phase of HEV-3ra or HEV4 infection showed hepatocyte degeneration, necrosis, swelling, and infiltration of inflammatory cells in the liver section and focal lymphocytic infiltration, connective tissue with hyalinization, and renal tubular arranged disorder in the kidney section. **(E)** The rabbit with HEV-3ra chronic infection showed hemorrhage, edema, and degeneration in the liver section. Focal lymphocytic infiltration, vertebral cell edema in renal medulla, and renal interstitium filled with protein effusion were seen in kidney tissue. **(F)** Group C serves as the control group for both HEV-3ra- and HEV4-infected groups. The liver and kidney sections from rabbits in control group C showed no visible pathological signs of HEV infection.

At the acute phase of HEV infection, focal lymphocytic infiltration and tubular protein casts were observed in the kidney, while hepatocyte degeneration and necrosis and collagen fiber hyperplasia in the portal area were seen in the liver section (**Figures 2A, B**). At the recovery phase, pathological lesions including focal lymphocytic infiltration, connective tissue with hyalinization, and renal tubular arranged disorder were seen in the kidney section, and degeneration, necrosis, and swelling were seen in the liver section (**Figures 2C, D**). At the chronic phase of HEV-3ra infection, pathological manifestations of focal lymphocytic infiltration, vertebral cell edema in renal medulla, and renal interstitium filled with protein effusion were observed in kidney tissue, while hemorrhage, edema, and degeneration were seen in the liver section (**Figure 2E**). No pathological lesions were observed in kidney or liver tissues in rabbits from the control group (**Figure 2F**).

**Figure 3:**
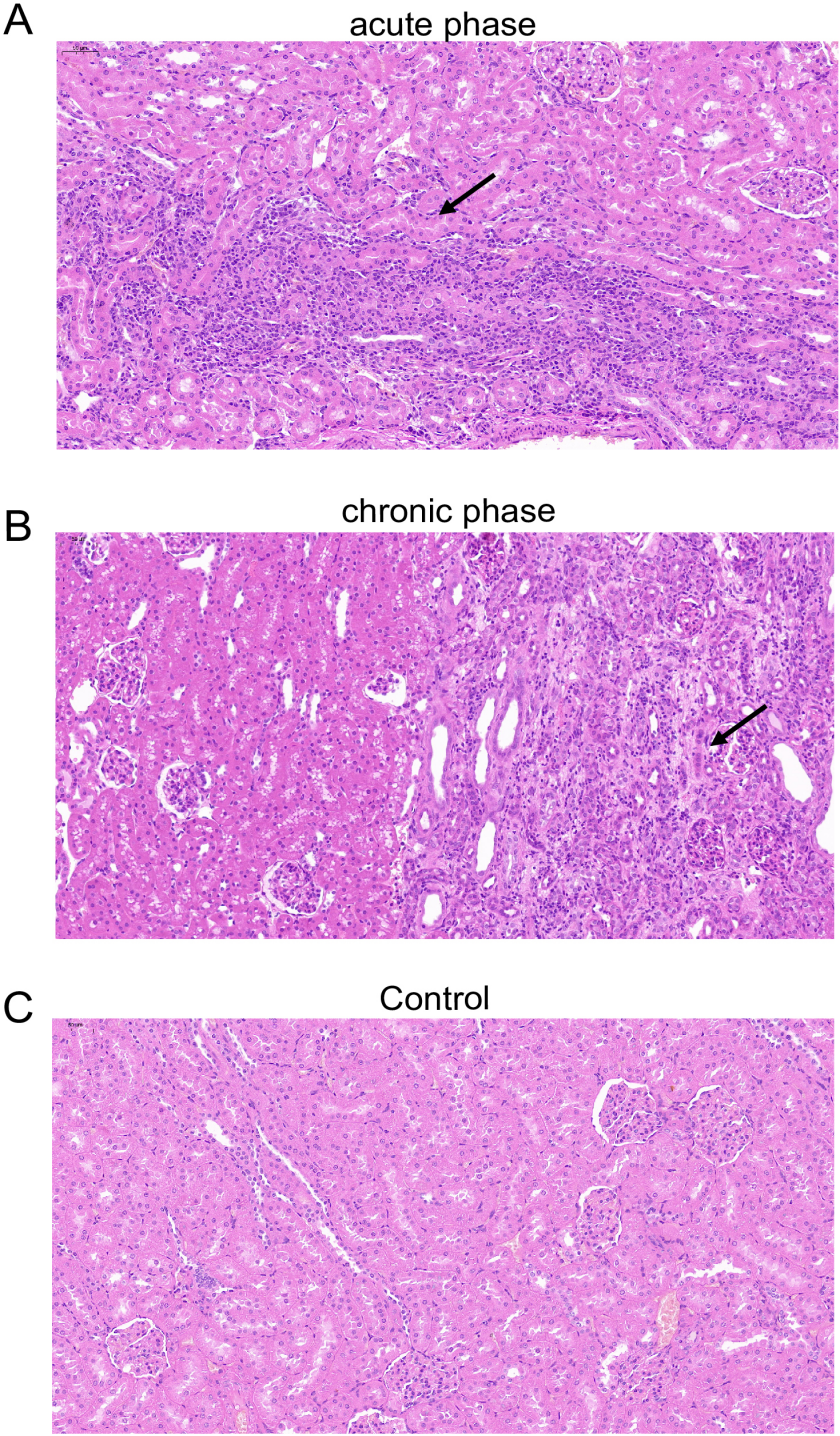
Histopathology of the kidney in an immunosuppressant-induced immunocompromised rabbit with HEV-3ra infection. **(A, B)** Focal lymphocytic infiltration, vertebral cell edema in renal medulla, and renal interstitium filled with protein effusion were seen in the kidney tissue in an immunosuppressant-induced immunocompromised rabbit with HEV infection at the acute phase **(A)** and the chronic phase **(B)**. **(C)** The kidney section of an immunosuppressant-induced immunocompromised rabbit without HEV infection showed no obvious pathological changes.

Corresponding to our AHE patient cohort, the rabbit in the current study was not treated with any immunosuppressants and presumed to be non-immunocompromised. We further investigate the kidney histopathology in an immunosuppressant-induced immunocompromised rabbit model with HEV-3ra infection as renal injury was described mostly in organ transplant patients who were receiving immunosuppressants. The kidney sections of these immunosuppressant-induced immunocompromised rabbit were from our previous studies (He et al., 2022; He et al., 2024). Compared to the immunosuppressant-induced immunocompromised rabbit without HEV-3ra infection, focal lymphocytic infiltration in the kidney, vertebral cell edema in renal medulla, and renal interstitium filled with protein effusion were seen at both acute and chronic phases in those with HEV-3ra infection (**Figure 3**).

## Discussion

Kidney disease related to HEV has been predominantly reported in immunosuppressed patients (Ali et al., 2001; Kamar et al., 2012; Taton et al., 2013; Del et al., 2015; Guinault et al., 2016). In the present study, non-immunocompromised AHE patients were enrolled, and 25% of them exhibited proteinuria, indicating HEV-associated renal injury. Previous studies have identified glomerular disease as the predominant manifestation in HEV-associated kidney diseases, including membranoproliferative glomerulonephritis, membranous glomerulonephritis, IgA-glomerulonephritis, and nephroangiosclerosis (Ali et al., 2001; Kamar et al., 2012; Taton et al., 2013; Del et al., 2015; Guinault et al., 2016). However, kidney biopsy was not performed in our cohort as all of the patients were diagnosed and treated only for acute hepatitis. It is noteworthy that kidney function test in HEV-infected non-immunocompromised patients may be frequently neglected, and a further diagnosis of kidney disease is impossible.

To further investigate the dynamics of kidney function during acute HEV infection and the viral replication in the liver and the kidney, which is difficult to conduct in clinical settings, we used a previously established rabbit model, which included HEV3 (rabbit-derived HEV-3ra) and HEV4 (human-derived HEV4) infection (Wang et al., 2017). Both serum ALT and Cr concentrations were elevated in all HEV-3ra-infected rabbits and a subset of HEV4-infected rabbits at the acute infection phase and returned to baseline level upon infection resolution. At the recovery phase, viral loads were significantly declined in the liver tissues compared to the acute phase but persisted at comparable levels in the kidney tissues in HEV-3ra-infected rabbits. These findings suggest that considerable amounts of HEV may harbor in the kidney at the recovery phase when HEV RNA in the liver dropped rapidly and was undetectable in the stool. This viral persistence may explain previous clinical observations where ~18% of chronic hepatitis E patients experienced viral relapse following ribavirin therapy cessation despite achieving undetectable HEV RNA in stool (Kamar et al., 2014). Thus, a solely negative HEV RNA result may be insufficient to conclude HEV clearance in some cases. The animal experiment together indicated a direct relationship between HEV infection and renal injury and suggested that the clearance of HEV might be protracted in the kidney. Therefore, patients with HEV-associated renal injury should be closely monitored even after fecal/serum HEV RNA is undetectable.

Accumulating evidence from animal models has documented HEV-associated renal pathology. In an HEV-infected cynomolgus monkey model, pathological changes including tubular protein casts and interstitial infiltration of inflammatory cells in the kidney were observed (Geng et al., 2016). Similarly, Mongolian gerbil models infected with HEV demonstrated glomerular atrophy, extension of the renal glomerulus capsular space, renal tubular epithelial cell swelling, degeneration, and necrosis, vacuolation of renal tubules, renal interstitial edema, and hemorrhage (Soomro et al., 2016). Both monkey and gerbil models were infected with HEV4, and renal injury was detected during the acute infection phase. The rabbit is another important animal model for HEV study (Liu et al., 2024; Liu et al., 2025). In this study, we systematically characterized kidney histopathology across multiple phases (including acute, recovery, and chronic phases) in both HEV3- and HEV4-infected rabbit models, respectively. Pathological changes were observed in the kidney, including lymphocytic infiltration, cell edema in renal medulla, and protein cast. The injury was more severe in rabbits infected with HEV-3ra than HEV4 overall. Notably, inflammation changes were more severe in the kidney of a rabbit with chronic HEV-3ra infection. These observations establish the rabbit model as a clinically relevant system to study HEV-associated renal injury across disease stages.

Our integrated analysis of biochemical and histopathological data revealed greater renal injury in HEV-3ra- versus HEV4-infected rabbits. This genotype-dependent pathogenicity aligns with clinical epidemiology: HEV3 accounts for most HEV-associated renal manifestations reported in human cases (Kamar et al., 2008a; Kamar et al., 2012; Taton et al., 2013; Leblond et al., 2024). Moreover, since rabbit itself is the natural host of HEV-3ra, the infectivity and replication of HEV-3ra in rabbit is more robust than that of the human HEV4 strain. This is also observed in our previous studies, including an HEV-infected pregnant rabbit model with adverse pregnancy outcomes (Li et al., 2019) and a chronic-HEV-infection-immunocompromised rabbit model with liver fibrosis (He et al., 2022).

HEV infection is strongly associated with glomerular diseases, and it is hypothesized that HEV-associated renal injury is due to viral antigen and antibody complex depositing in the glomerulus, similar to hepatitis-C-virus-associated renal diseases (Ozkok et al., 2014; Pol et al., 2019). In fact, several studies have suggested a large amount of circulating non-infectious HEV viral antigens (the secreted form of HEV ORF2 protein) in the serum and found to be enriched in the urine and in kidney tissues (Marion et al., 2019; He et al., 2022; Ying et al., 2022). A recent study further discovered that IgG/HEV ORF2 protein immune complexes are extensively deposited in the glomerulus, which linked the secreted form of HEV ORF2 protein with renal injury (Leblond et al., 2024). Further investigation is warranted to elucidate the precise immune-mediated mechanisms underlying HEV-associated renal injury.

Our study has several limitations. First, the genotype of HEV infected in these AHE patients was not determined as HEV RNA testing and sequencing are still not routine tests in most hospitals in China. Second, only a rabbit model was used in the current study. Systematic investigations across diverse animal models are warranted to better substantiate the generalized impact of HEV on renal pathophysiology.

In summary, our study demonstrates that HEV-associated renal injury is not uncommon in non-immunocompromised AHE patients. We also demonstrated that HEV clearance was protracted in the kidney even as the virus stopped shedding in the stool in a rabbit model, underscoring the necessity of extended virological monitoring for patients with HEV-associated renal injury even after the achievement of stool HEV RNA negativity to mitigate relapse risk. These findings identify the kidney as a potential extra-hepatic reservoir and persistent viral replication site during HEV infection.

## Data Availability

The original contributions presented in the study are included in the article/supplementary material. Further inquiries can be directed to the corresponding authors.
